# Complex patterns of reticulate evolution in opportunistic weeds (*Potentilla* L., Rosaceae), as revealed by low-copy nuclear markers

**DOI:** 10.1186/s12862-020-1597-7

**Published:** 2020-03-18

**Authors:** Nannie L. Persson, Torsten Eriksson, Jenny E. E. Smedmark

**Affiliations:** Department of Natural History, University Museum, University of Bergen, Postboks 7800, N-5020 Bergen, Norway

**Keywords:** Low-copy nuclear markers, Hybridization, Ivesioids, Molecular cloning, Molecular phylogeny, Polyploidy, *Potentilla*, Reticulate evolution

## Abstract

**Background:**

Most cinquefoils (*Potentilla* L., Rosaceae) are polyploids, ranging from tetraploid (4*x*) to dodecaploid (12*x*), diploids being a rare exception. Previous studies based on ribosomal and chloroplast data indicated that Norwegian cinquefoil (*P. norvegica* L.) has genetic material from two separate clades within *Potentilla*; the Argentea and the Ivesioid clades – and thus a possible history of hybridization and polyploidization (allopolyploidy). In order to trace the putative allopolyploid origin of the species, sequence data from low-copy, biparentally inherited, nuclear markers were used. Specimens covering the circumpolar distribution of *P. norvegica* and its two subspecies were included, along with the morphologically similar *P. intermedia*. *Potentilla* species of low ploidy level known to belong to other relevant clades were also included.

**Results:**

Gene trees based on three low-copy nuclear markers, obtained by Bayesian Inference and Maximum Likelihood analyses, showed slightly different topologies. This is likely due to genomic reorganizations following genome duplication, but the gene trees were not in conflict with a species tree of presumably diploid taxa obtained by Multispecies Coalescent analysis. The results show that both *P. norvegica* and *P. intermedia* are allopolyploids with a shared evolutionary history involving at least four parental lineages, three from the Argentea clade and one from the Ivesioid clade.

**Conclusions:**

This is the first time that reticulate evolution has been proven in the genus *Potentilla*, and shows the importance of continuing working with low-copy markers in order to properly resolve its evolutionary history. Several hybridization events between the Argentea and Ivesioid clades may have given rise to the species of Wolf’s grex Rivales. To better estimate when and where these hybridizations occurred, other Argentea, Ivesioid and Rivales species should be included in future studies*.*

## Background

The evolution of species is usually considered to be a slow process, working over thousands or even millions of years. Sometimes, however, new species evolve within a relatively short period of time through polyploidization. This phenomenon is common throughout the vascular plants, where genome duplications can be found from the ferns [1] and lycopods [2], to the asterids [3]. Two main types of polyploidization are recognized; autopolyploidization, where the duplication occurs within a single species, and allopolyploidization, where the duplication occurs in combination with hybridization between two different species [4]. A doubling of the chromosomes can make a sterile hybrid fertile [5, 6] and cause a reproductive barrier between individuals of the new genomic state and the old state [6, 7]. This may create a new, independently evolving, lineage that could thus be regarded as a new species [8].

The rose family (Rosaceae Juss.) is well known for its many polyploid taxa, and there seem to have been a large number of independent auto- and allopolyploidization events during its evolutionary history [9–11]. Chromosome counting data, summarized by Vamosi and Dickinson [12], suggest that around half of the family’s genera include at least one polyploid species. Some, as for instance *Acaena* L., *Alchemilla* L. and *Sorbaria* (Ser.) A. Braun, consist only of polyploids.

The cinquefoils, *Potentilla* L.*,* is an example of a genus in Rosaceae with mixed ploidy levels. According to the Chromosome Counts Database [13] only a few species seem to be exclusively diploid, e.g. *P. biflora* Willd. ex Schltdl., *P. freyniana* Bornm. and *P. valderia* L*.* At the other end, *P. gracilis* Douglas ex Hook., *P. tabernaemontani* Asch. and *P. indica* (Jacks.) Th. Wolf have been reported to have dodecaploid (12*x*) populations. Furthermore, it is not uncommon for single species to have multiple ploidy levels. The genus has undergone a major recircumscription since the first molecular studies of the group were performed [14, 15]; both plastid and nuclear ribosomal markers showed that it had been polyphyletic. They strongly indicated that some previous *Potentilla* species are more closely related to the strawberries, *Fragaria* L., in the Fragariinae clade, such as those species now assigned to the genera *Dasiphora* Raf. and *Drymocallis* Fourr. In contrast, the genus *Duchesnea* Sm. and some species of *Sibbaldia* L., were instead shown to belong to *Potentilla* [14, 16]. However, the debate on where to draw the generic delimitation is still ongoing; as whether to include the genus *Argentina* Hill. and its sisters [15, 17] or not [18–20]. Regardless whether *Argentina* is included or not, the genus is still polyphyletic in certain classifications where *Duchesnea* (*P. indica*) and the genera of the North American Ivesioid clade (*Horkelia* Cham. & Schltdl., *Horkeliella* (Rydb.) Rydb. and *Ivesia* Torr. & A.Gray) are separated from *Potentilla* [17, 21, 22]. Within *Potentilla* in the strict sense, there are a number of well supported subclades, such as the Alba, Reptans and Ivesioid clades [23]. The most species-rich subclade, called either “Argentea” [23] or “core group” [18] in previous studies, is, however, in itself poorly resolved [18, 20, 23].

Previous studies have found a possible connection between the Argentea and Ivesioid clades in the polyploid species *P. norvegica* L. This species has been shown to have different phylogenetic relationships depending on whether the analyses were based on chloroplast [15, 18, 23] or nuclear ribosomal data [14, 15, 23]; with chloroplast data the species groups with the Argentea clade, but with ribosomal data it groups with the Ivesioids. Töpel et al. [23] speculated that this may be due to an evolutionary history of polyploidization in combination with hybridization between these two clades. It is, however, not previously known to what extent these two processes have played a part in the formation of *P. norvegica*, or if the discordance between chloroplast and ribosomal data is the result of other processes, such as a single hybridization event followed by introgression [24].

In his monograph of *Potentilla*, Wolf [25] placed *P. norvegica* together with 20 other species in his “grex” Rivales. Of these, *P. intermedia* L. and *P. supina* L. have a similar circumpolar distribution as *P. norvegica,* while the North American species *P. biennis* Greene and *P. rivalis* Nutt. are morphologically similar to *P. norvegica*. Another common feature is that they are annuals or short-lived perennials [17, 25]. *Potentilla norvegica* was originally described by Linnaeus [26] as two separate species based on stem and leaflet morphology of European specimens; *P. norvegica* L. and *P. monspeliensis* L. In 1803, Michaux [27] described *P. hirsuta* Michx. based on North American specimens, but Ledebour [28] later synonymized *P. monspeliensis* and *P. hirsuta* under *P. norvegica*. Nevertheless, there is striking morphological variation within the species, and today two subscpecies are generally accepted. However, it has been unclear which subspecies name has priority. In 1904, Ascherson and Graebner [29] described “*P. norvegica* II. *monspeliensis*”, by some nomenclatural databases interpreted as a subspecies [30, 31]. However, Hylander [32] must have interpreted this as a variety. Since names only have priority at the same nomenclatural rank [33], he was able to list “II. *monspeliensis”* under *P. norvegica* ssp*. hirsuta* (Michx.) Hyl. The name that will be used in this study is therefore *Potentilla norvegica* ssp*. hirsuta*, which refers to specimens displaying the morphology first used to describe *P. monspeliensis*. Since *P. norvegica* ssp*. hirsuta* is the most common subspecies in North America, it is sometimes referred to as the American form, and the autonym ssp*. norvegica* as the European form, but there are numerous findings of ssp*. hirsuta* in Europe. Most floras argue for an East European origin of the species, and that ssp*. hirsuta* later has dispersed to Europe from North America [34–38]. However, no molecular phylogenetic work has been performed in order to test these hypotheses.

The two types of molecular data most commonly used in phylogenetic studies of plants both have the inconvenience that they are not able to detect reticulate patterns in phylogenetic trees. The chloroplast is inherited uniparentally and nuclear ribosomal markers are most often subject to concerted evolution, while low-copy nuclear markers are inherited biparentally and present in each subgenome after a polyploidization event [39]. This means that they have the potential to retrieve polyploid signals in a single gene tree. For instance, Smedmark et al. [40] resolved the Colurieae clade in Rosaceae with its many polyploid species using this type of marker. However, different gene trees do not necessarily depict the same evolutionary history, due to processes such as horizontal gene transfer, deep coalescence and lineage sorting [41]. Furthermore, since it is not possible to know beforehand which sequences are homologous, low-copy markers cannot be concatenated to form larger datasets. Therefore, when polyploidy is present, it is important to investigate several low-copy markers in order to find the species tree. In a phylogenetic gene tree covering a simple polyploidization event, the gene copies of an autopolyploid (paralogues) would be each other’s sisters, while the gene copies of an allopolyploid (homoeologues) would be sisters to their respective parental lineage. This has a number of effects on species trees, since the evolutionary history of an allopolyploid would be better represented by a reticulate pattern where lineages merge, rather than by a traditional bifurcating tree [24].

By using low-copy nuclear markers, this study aims to determine (1) if *Potentilla norvegica* and *P. intermedia* have an allopolyploid evolutionary history resulting from hybridization between the Argentea and Ivesioid clades; (2) if this is the case, do they share polyploidy events; and (3) if morphology and geography are concordant with intraspecies phylogeny in *P. norvegica*.

## Results

### Sequence alignment

**Fig. 1:**
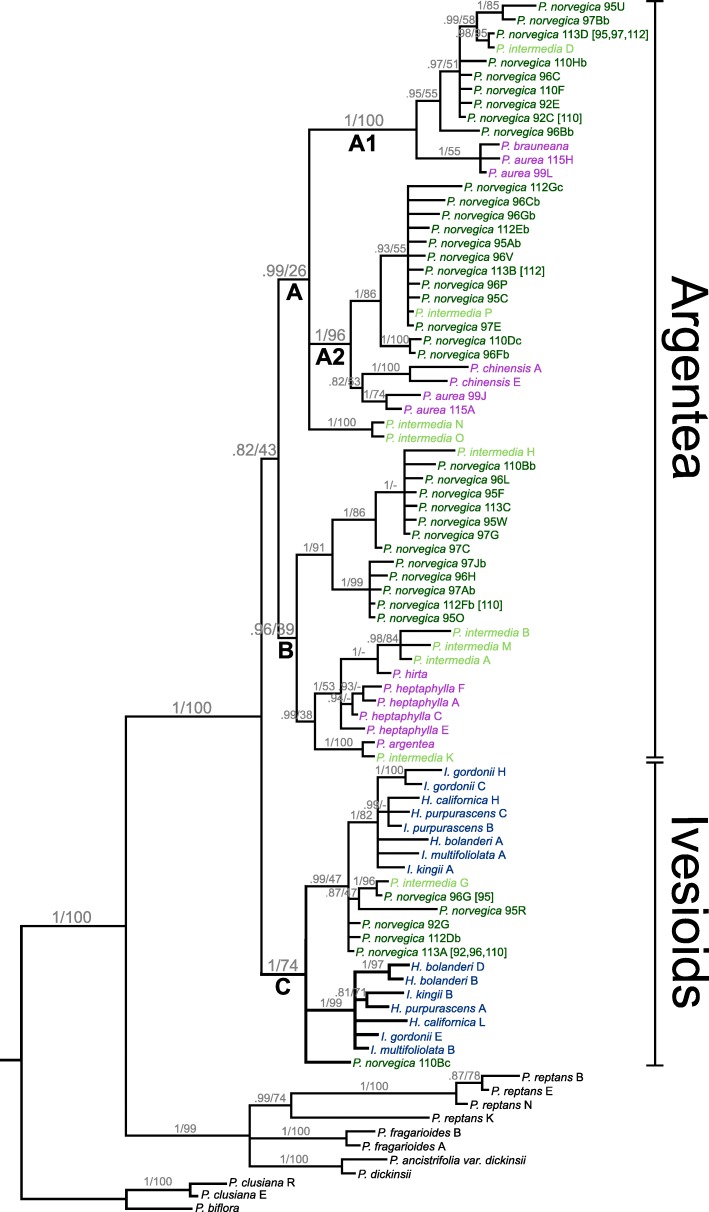
Bayesian 80% majority rule consensus tree of the GAPCP1 gene in *Potentilla*. Support values are shown on the branch below the corresponding nodes: Bayesian Inference posterior probabilities to the left, and Maximum Likelihood bootstrap values to the right of the slashes. Clades discussed in the text are marked with letters (and numbers). The extent of the Argentea clade and the Ivesioid clade is noted to the right. Species name suffixes indicate individuals and letters indicate clones (cf. Table 2). Species name colours: Dark green – *P. norvegica*; light green – *P. intermedia*; blue – Ivesioid species; purple – Argentea species

**Fig. 2:**
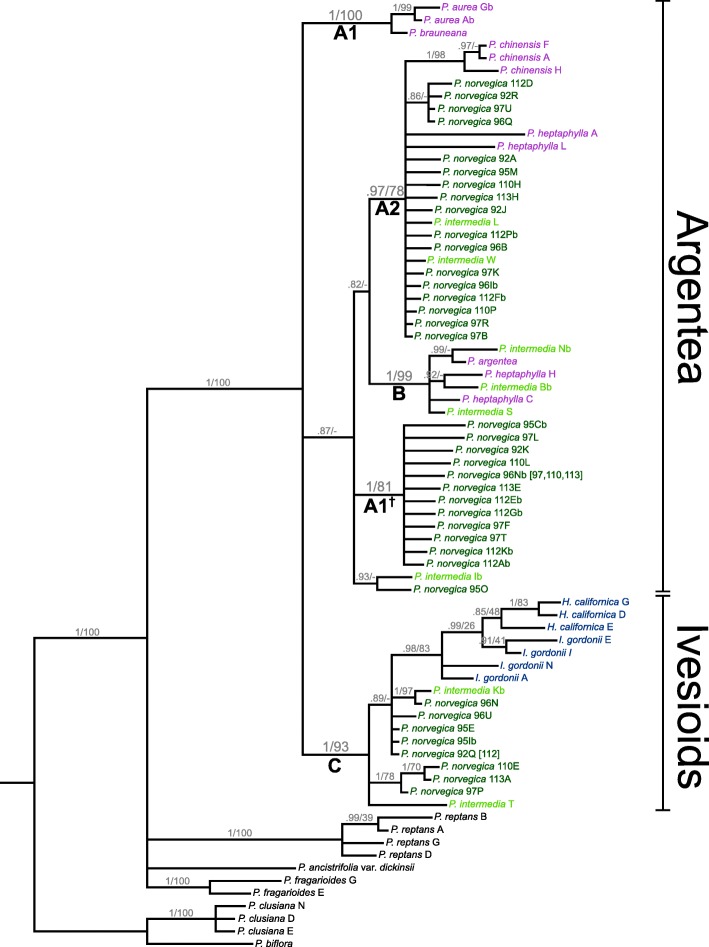
Bayesian 80% majority rule consensus tree of the GBSSI-1 gene in *Potentilla*. Support values are shown on the branch below the corresponding nodes: Bayesian Inference posterior probabilities to the left, and Maximum Likelihood bootstrap values to the right of the slashes. Clades discussed in the text are marked with letters (and numbers). The extent of the Argentea clade and the Ivesioid clade is noted to the right. Species name suffixes indicate individuals and letters indicate clones (cf. Table 2). Species name colours: Dark green – *P. norvegica*; light green – *P. intermedia*; blue – Ivesioid species; purple – Argentea species. † This clade of *P. norvegica* sequences resolved with *P. aurea* and *P. brauneana* in the ML analysis (bs 66)

**Fig. 3:**
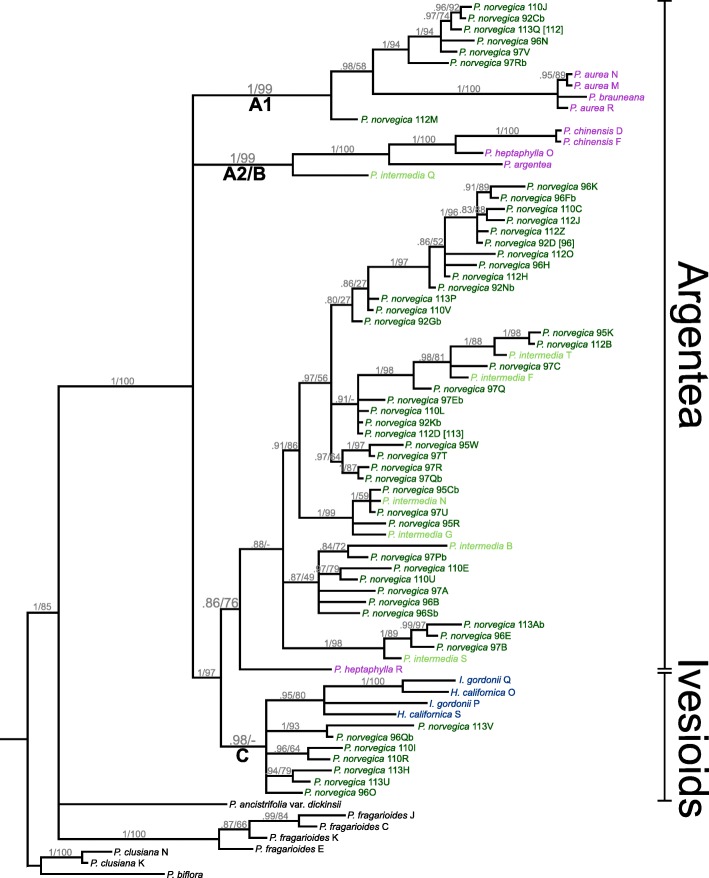
Bayesian 80% majority rule consensus tree of the DHAR2 gene in *Potentilla*. Support values are shown on the branch below the corresponding nodes: Bayesian Inference posterior probabilities to the left, and Maximum Likelihood bootstrap values to the right of the slashes. Clades discussed in the text are marked with letters (and numbers). The extent of the Argentea clade and the Ivesioid clade is noted to the right. Species name suffixes indicate individuals and letters indicate clones (cf. Table 2). Species name colours: Dark green – *P. norvegica*; light green – *P. intermedia*; blue – Ivesioid species; purple – Argentea species

All markers shared some identical *Potentilla norvegica* sequences across individuals, which are marked in brackets in the gene trees (Figs. 1, 2 and 3).

In addition, two GAPCP1 sequences from *P. intermedia* were identical to two *P. norvegica* sequences (P to 97E and D to 113D), while the GBSSI-1 *P. intermedia* sequence Kb and *P. norvegica* sequence 96N differed in only one base pair.

### Phylogenetic analyses

#### Partitioning and model suggestions

The lowest log likelihood value for the partitioning and model analyses were obtained under the AICc criterium for all markers. Partitioning schemes and their assigned models are found in Table 1.

**Table 1:** Partitioning and evolutionary models used for analysis in MrBayes, as suggested by PartitionFinder2

**GAPCP1**				
Subset	1st codon	2nd codon	3rd codon	introns
Model	F81 + G	JC	GTR + G	HKY + G
**GBSSI-1**				
Subset	1st codon	2nd codon	3rd codon + introns	
Model	GTR + I + G	JC + I	GTR + I + G	
**DHAR2**				
Subset	intron			
Model	HKY + I + G			

#### Bayesian and ML analyses

The Bayesian phylogenetic analysis of GAPCP1 resolved *Potentilla norvegica* sequences in four clades (Fig. 1). Three of these clades were sisters to Argentea species (clade A1, posterior probability 1.0; A2, pp. 1.0; B, pp. 0.96) and one was sister to the Ivesioids (C, pp. 1.0). *Potentilla intermedia* was found in the same four clades. The A1 and A2 clades formed a polytomy together with two *P. intermedia* sequences (A, pp. 0.99). The node connecting the A and B clades, i.e. corresponding to the Argentea clade, was not strongly supported (pp 0.82). The Ivesioid genera (*Horkelia*, *Horkeliella* and *Ivesia*) in clade C were divided into two subclades (both pp. 1.0), with at least one sequence from each species in each subclade. The Maximum Likelihood analysis showed the same topology, but only clades A1 and A2 were supported (bootstrap support 100 and 96, respectively).

The Bayesian analysis of GBSSI-1 showed *P. norvegica* sequences in three clades (Fig. 2), of which two correspond to A2 (pp 0.97) and C (pp 1.0) in the GAPCP1 tree. There was, however, no *P. norvegica* homoeologue associated with the Argentea species in clade B (pp 1.0). *Potentilla intermedia* homoeologues were found in clades A2, B and C. Clades A2 and B were sisters with low support (pp 0.82). They formed a polytomy (pp 0.87) with the third *P. norvegica* clade (pp 1.0) and a small clade consisting of one *P. norvegica* and one *P. intermedia* sequence (pp. 0.93). This polytomy was in turn in a polytomy (pp 1.0) with clade C and the Argentea species from clade A1 (pp 1.0). Thus, there was no Argentea clade in this tree. Within clade C, the Ivesioid species formed one subclade (pp 0.98), in which two of the four *Ivesia* sequences were sisters to *Horkelia* (pp 0.99), while the other two were unresolved. The ML analysis showed clades A1 (bs 66), A2 (bs 78), B (bs 99) and C (bs 93), but their relative positions were not supported. The clade with only *P. norvegica* sequences, present in the Bayesian tree, was placed as sister to *P. aurea* and *P. brauneana* (A1) in the ML tree*.* Even though bootstrap support was low, we will refer to this *P. norvegica* clade as A1^†^.

The Bayesian analysis of DHAR2 (Fig. 3) also showed *P. norvegica* in three clades, two of them corresponding to A1 (pp 1.0) and C (pp 0.93) in the other trees, while the third had not been seen previously. This clade consisted of *P. norvegica*, *P. intermedia* and one *P. heptaphylla* sequence, and was supported as sister to clade C (pp 1.0), while the clade itself had low support (pp 0.86). There was no supported Argentea clade in this tree. The Ivesioids formed one subclade in clade C, where one of two *Horkelia* sequences and one of two *Ivesia* sequences were sisters (pp 1.0), while the other two were unresolved. The ML analysis showed no conflicting topology of the major clades, but there were two Ivesioid subclades (bs 83 and 100), with one *Ivesia* and one *Horkelia* sequence in each, and those were supported as sisters (bs 80). The sister clade to clade C was also supported (bs 76).

No clade was specific to, or missing, any of the two *P. norvegica* subspecies or seven individuals throughout all three gene trees. For instance, clade C was missing individual 97 in the GAPCP1 tree and individuals 92, 95, 97 and 112 in the DHAR2 tree, while all individuals were represented in this clade in the GBSSI-1 tree.

Five species with previously published diploid chromosome counts [13], *P. aurea*, *P. chinensis*, *P. clusiana*, *P. fragarioides* and *P. heptaphylla*, failed direct sequencing and were therefore molecularly cloned. In the GBSSI-1 and DHAR2 trees, *P. aurea* was sister to *P. brauneana* in clade A1 (pp 1.0). However, in the GAPCP1 tree two *P. aurea* sequences were placed in clade A1, but the other two were placed in clade A2 as sisters to *P. chinensis* (pp 0.82). In the GAPCP1 tree, all *P. heptaphylla* sequences were placed in clade B, but in the GBSSI-1 tree two sequences were found in A1 and two found in A2. In the DHAR2 tree they were even further apart, with one sequence as sister to *P. chinensis* in A2/B and one as sister to *P. norvegica* and *P. intermedia* in the sister clade to clade C. The sequences of *P. chinensis*, *P. clusiana* and *P. fragarioides* formed clades of their own.

#### Control analyses

The control ML analyses for putatively missed *P. norvegica* gene copies did not reveal any new clades or overlooked patterns in terms of subspecies or geographical origin. However, two excluded *P. intermedia* GBSSI-1 sequences were indicated to belong in clade A1. One of these was added to the dataset, but the Bayesian analysis resulted in the collapse of clades B and C, which received high support in the other trees. Similarly, one *P. intermedia* DHAR2 sequence was indicated to belong in clade C, but when added to the dataset it also resulted in the collapse of several clades. Both sequences were therefore excluded again from their respective datasets.

#### Multispecies coalescent analysis

**Fig. 4:**
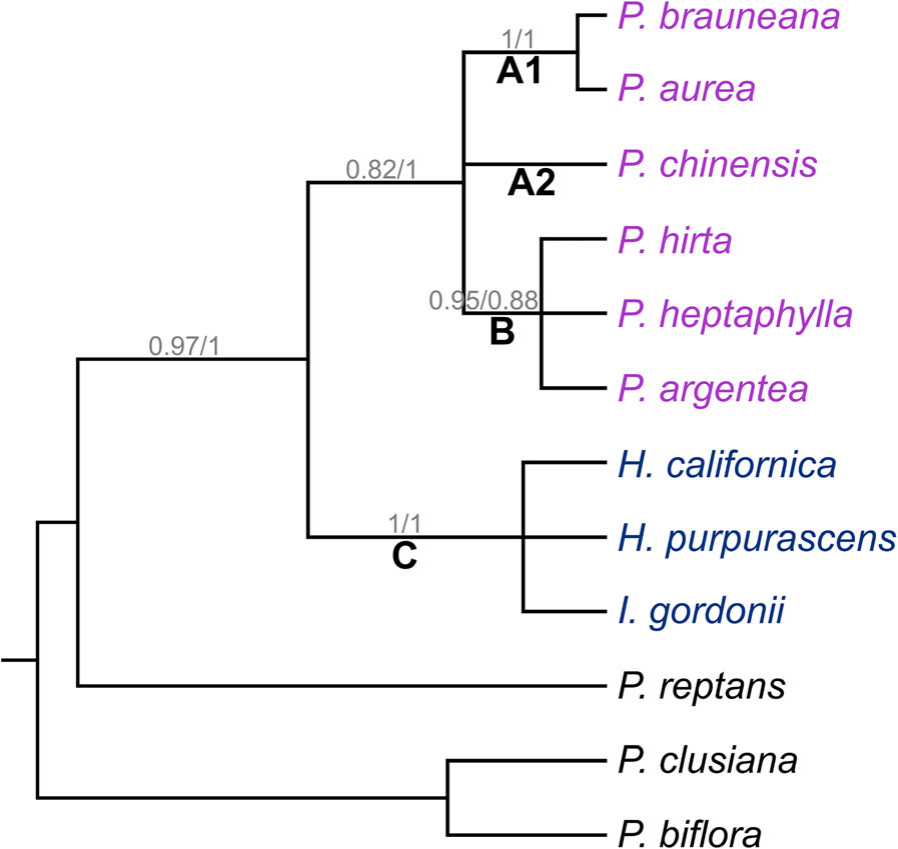
Bayesian 80% majority rule consensus tree from the multispecies coalescent analysis. Support values are shown on the branch below the corresponding nodes: Posterior probabilities from the analysis of low-copy markers only are shown to the left of the slashes, and those from the analysis including both low-copy and chloroplast markers are shown to the right. Clades discussed in the text are marked with letters (and numbers). Species name colours: Blue – Ivesioid species; purple – Argentea species

The substitution model suggested for all markers was HKY [42], with gamma as site heterogeneity model for GAPCP1 and GBSSI-1, and invariant sites for DHAR2. The clock model and tree prior that was best fitted to the low-copy marker only dataset was a relaxed uncorrelated lognormal clock with a birth-death process, and for the combined low-copy and chloroplast marker dataset a relaxed uncorrelated lognormal clock with a birth process. The two trees had the same topology, but some of the support values differed (Fig. 4). In both trees, the Ivesioid clade was supported (pp 1.0) and *P. aurea* and *P. brauneana* were sisters (pp 1.0), corresponding to clade A1 in the gene trees. *Potentilla hirta*, *P. heptaphylla* and *P. argentea* formed a polytomy (pp 0.95 in the low-copy marker dataset and pp. 0.88 in the combined dataset) corresponding to clade B, while *P. chinensis* of clade A2 was unresolved. The Argentea clade received low support (pp 0.82) in the low-copy marker tree and full support (pp 1.0) in the combined tree.

### Morphological study

Most specimens studied from the collections of BG, GB, O, S and UPS were of intermediate morphology. They had, for instance, whole stipules (ssp*. norvegica*), but obovate leaflets and obtuse leaflet teeth (ssp*. hirsuta*). For European specimens, there was approximately equal occurrence of typical individuals of the two subspecies. For the North American and East Russian specimens, typical individuals showing the ssp*. hirsuta* morphology were more common than those showing the ssp*. norvegica* morphology. The few North American specimens showing the ssp*. norvegica* morphology were all but one (Alaska, USA) collected in the East (Ontario, Canada, to New York, USA), a pattern also seen by Rydberg [43].

## Discussion

Despite the slightly different topologies of the three single-copy nuclear markers presented in this study, it is clear that both *Potentilla norvegica* and *P. intermedia* are allopolyploids with a shared evolutionary history involving one parental lineage in the Ivesioid clade and multiple parental lineages in the Argentea clade. These results rule out a simple case of introgression, and reveal a complex reticulate evolutionary history of several hybridization events in combination with polyploidization. For *P. norvegica*, there was no condordance between geography and intraspecies phylogeny. Thus, on the basis of our data we see no support for species differentiation, as first suggested by Linnaeus [26], since the majority of the individuals studied in the herbaria were of intermediate morphological form. Neither did our molecular data support a division into subspecies, but a more extensive study involving more individuals of especially ssp*. norvegica* would be better able to investigate the relationship between them.

As previously shown in studies based on chloroplast and ribosomal data [14, 15, 18, 20, 23, 44], the Ivesioid clade is deeply nested in *Potentilla* (Figs. 1, 2 and 3). Thus, following the established practice of only recognizing monophyletic taxa, the Ivesioid genera *Horkelia*, *Horkeliella* and *Ivesia* should be incorporated in *Potentilla*. The type species of *Potentilla*, *P. reptans*, is part of the small Reptans clade, which is the sister clade to the Argentea and Ivesioid clades. If the Ivesioid genera were to be retained, the many species of the large Argentea clade would have to be reclassified, and it is probable that almost all would have to change names. However, the new evidence presented here of a hybridization event between the Argentea and Ivesioid clades indicate a close relationship between the groups, and adds a compelling argument for including the Ivesioid genera in *Potentilla*.

The three gene trees conform well to the backbone reference (Fig. 4), apart from some *P. aurea* and *P. heptaphylla* sequences. It is, however, clear that one *P. norvegica* GBSSI-1 homoeologue (subgenome-specific gene copy) is missing in clade B and one *P. intermedia* GBSSI-1 homoeologue is missing in clade A1^†^ (Fig. 2). In the DHAR2 tree (Fig. 3), there is a major rearrangement in which the Ivesioid clade C is sister to what could be assumed to be parts of clade A2 or B. In addition, contrary to previous analyses based on chloroplast and nuclear ribosomal data [16, 18, 20, 23], the support for the Argentea clade was low both in the individual gene trees (Figs. 1, 2 and 3) and in the species tree based on low-copy markers only (Fig. 4). Thus, it is evident that phylogenetic relationships of low-copy nuclear genes are complicated by a number of evolutionary processes. A polyploid genome with high genetic redundancy may be subjected to large genomic alterations, such as deletions, insertions, or recombinations, to a high extent without causing fatal effects [45]. For instance, entire homoeologues may be lost as a response to genomic reorganization after polyploidization [46, 47] or via incomplete lineage sorting during speciation after hybridization [41]. Furthermore, if an interallelic recombination [24] splits a gene in two unequal parts during meiosis, the new recombinant will position itself as sister to its major donor in the gene tree, and such a process might explain the clade rearrangement seen in the DHAR2 treee.

Previous dating analyses have assigned somewhat different ages to the *Potentilla* crown group (excluding *Argentina*), either between ca 36 to 15 Mya [18, 20, 48] or between ca 56 to 32 Mya [44]. Estimations of the Agentea-Ivesioid split also varies, with ages between 15.2–9.8 Mya [18, 48] and 36.6–18.7 Mya [44]. There is also disagreement as to whether the Argentea crown clade is younger [44] or older [18, 48] than the Ivesioid crown clade, but this may be a sampling issue since undersampling of a species rich sister clade would tend to result in underestimating the age of its crown. Today, the Argentea clade consists of the majority of the *Potentilla* species. They have a circumpolar distribution in the Northern Hemisphere, are adapted to a variety of climates, and are of multiple ploidy levels. In contrast, the Ivesioids are limited to dry areas in western United States [21] and are, as far as known, tetraploid [13]. According to Töpel et al. [44] they also evolved in the same area, while Dobeš and Paule [18] estimated an origin in East Asia both for the *Potentilla* crown group and the Ivesioids. However, considering the Ivesioids being geographically restricted and ecologically specialized, the Western American origin of the crown clade found by Töpel et al. [44] may be the most plausible. It is notable, however, that if they are indeed sister groups, their stem lineages are of the same age, and any species that would fall below the crown clades of Argentea or the Ivesioids are either unsampled or extinct.

During the Eocene (56–33.9 Mya [49]), before or in the early stages of the diversification of the *Potentilla* crown group, the North Atlantic land bridge was broken up [50, 51] and the Turgai strait still separated Asia from Europe [51]. A land bridge over the Bering strait existed during most of the later Tertiary to mid Pliocene [51–53], and the original dispersal of the Ivesioid and Argentea ancestors from Asia to North America is most likely to have occurred before its breakup. Today the Bering Strait area is subject to very cold and long winters, but the clade ages suggested by Töpel et al. [44] indicate that the dispersal may have coincided with the Mid Miocene Climatic Optimum, when the Earth was on average 3 °C warmer than present [54]. However, considering the current cold climate tolerance of both *P. norvegica* and *P. intermedia* [17, 38], dispersal did not necessarily have to have coincided with warmer periods. Therefore, the younger clade ages estimated by Dobeš and Paule [18] and Feng et al. [20] need not be dismissed.

Regardless of their relative ages, and judging from extant species, the Argentea clade has gone through many more speciations, polyploidizations and hybridizations than the Ivesioid clade. Nonetheless, there is an indication of an early autopolyploid event in the Ivesioids, and this is especially evident in the GAPCP1 tree (Fig. 1); the two subclades in clade C each contain one or two sequences of all Ivesioid species included.

**Fig. 5:**
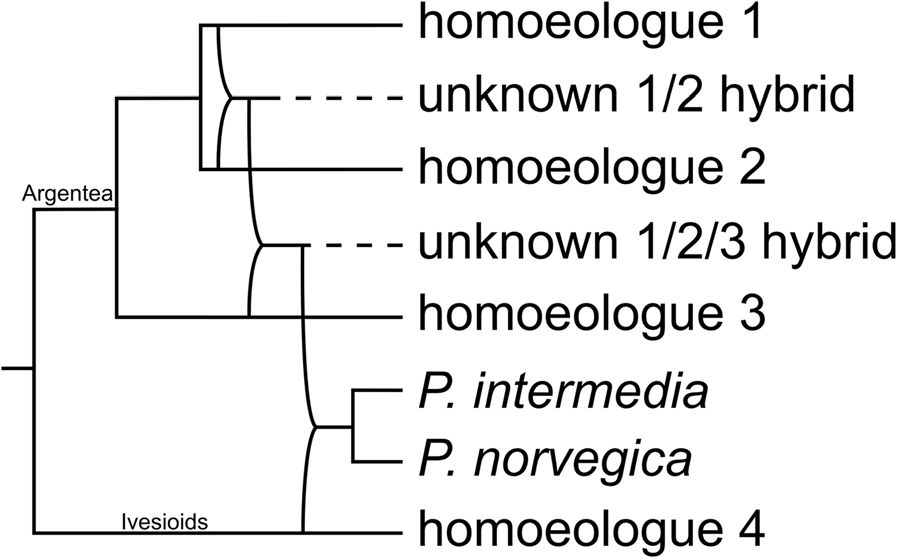
Manually constructed reticulate tree based on our interpretation of the homoeologues in the GAPCP1 gene tree. The tree shows one hypothesis of the events needed for the four GAPCP1 homoeologues to end up in *Potentilla norvegica* and *P. intermedia*

The single *P. norvegica* homoeologue in clade C (Figs. 1, 2 and 3) indicates that the Argentea-Ivesioid hybridization may have happened before polyploidization and diversification of the Ivesioid crown group. This makes the hybridization event difficult to pinpoint geographically; Töpel et al. [44] predicted a wide climate preference for the Ivesioid ancestor, and both *P. norvegica* and *P. intermedia* have weedy growth habits and can be found all around the Northern Hemisphere. Neither is it possible to say, based on our species sample and the resolution of our gene trees, if the Argentea-Ivesioid hybridization is the oldest or the most recent. To illustrate the mode of speciation that *P. norvegica* and *P. intermedia* have gone through, one possible chain of events is shown in Fig. 5 based on our interpretation of the GAPCP1 tree.

The phylogenetic pattern seen in chloroplast markers of Rivales species (in the sense of Wolf [25]) occurring in North America suggests that other species than *P. norvegica* and *P. intermedia* may have connections to the Argentea-Ivesioid hybridization event [18, 44]. Based on chloroplast data, *P. norvegica*, *P. newberryi*, *P. rivalis* and *P. supina* resolve with the Argentea clade, while *P. biennis* is sister to the Ivesioid clade. For *P. norvegica*, it is evident that the pollen donor came from the Ivesioid clade [23], and therefore it is notable that *P. biennis* is the only Rivales species that resolves with the Ivesioid clade. *Potentilla biennis*, *P. newberryi* and *P. rivalis* have a limited central to western North American distribution similar to that of the Ivesioids [17]. In addition, *P. biennis* and *P. rivalis* are morphologically similar to *P. norvegica*. Thus, it seems likely that the Argentea-Ivesioid hybridization event occurred in North America rather than in Asia. That would make the Eastern European origin of *P. norvegica*, as proposed by many floras [35, 37, 38], doubtful. It is therefore possible that the Rivales group originated following multiple hybridization events between the two clades. To better pinpoint where they occurred and which evolutionary routes that were then taken by the lineages that emerged, additional Argentea and Rivales species of various ploidy levels should be included in future analyses, such that all continents are better covered.

The four homoeologues that were found in *P. norvegica* had a high degree of variation. In the case of *P. intermedia*, this variation seemed even greater, since it is found in more subclades than *P. norvegica*. Both *P. norvegica* and *P. intermedia* have more than one ploidy level reported [13], and there are many other examples of plant populations with mixed ploidy levels [42, 55–57]. Sterile hybrids may still be able to produce offspring through apomixis, and this apomixis is in turn heritable [58]. According to Asker [59], both *P. norvegica* and *P. intermedia* can reproduce in this manner, which could explain the existence of multiple ploidy levels and high sequence variation within the two species. In addition, several of the putatively diploid species (*P. aurea, P. chinensis*, *P. clusiana*, *P. fragarioides* and *P. heptaphylla*) [13] included in this study failed direct sequencing of all markers, and showed a remarkable sequence variation. *Potentilla heptaphylla* was resolved together with *P. argentea* and *P. hirta* in the backbone reference (pp 0.95/0.88) (Fig. 4), but was seen in three different clades (A2, B and C) in the separate gene trees. This suggests allopolyploidy rather than single gene duplications, since the gene copies were resolved as sisters to different species in the same gene tree. The ploidy level of *P. aurea* is difficult to determine solely from the results presented here, since it is found in clades A1 and A2 in the GAPCP1 tree, but only in clade A1 in the GBSSI-1 and DHAR2 trees. However, as seen for *P. norvegica* in the GBSSI-1 and DHAR2 trees, it is possible that *P. aurea* and *P. heptaphylla* have lost homoeologues too. Future studies of polyploid species in *Potentilla* should consider chromosome counting and flow cytometry of the specimens included in order to more securely connect the gene trees with ploidy level, in addition to recreate a more accurate, reticulate species tree.

## Conclusions

This is the first study of species level relationships and reticulate patterns in *Potentilla* based on low copy nuclear markers. With this type of data it was possible to reveal a complex evolutionary history of polyploidizations and hybridizations, not only within previously identified subclades, but also between subclades. The nature of the results, and implications for the interpretation of evolutionary events and distribution patterns, demonstrate the importance of continued work with this kind of data.

The gene trees showed that *P. norvegica* and *P. intermedia* are allopolyploids with multiple parental lineages in the Argentea clade, and one in the Ivesioid clade. This close relationship between the two clades is one of several arguments for an inclusion of the genera of the Ivesioid clade (*Horkelia*, *Horkeliella* and *Ivesia*) in *Potentilla*. This inclusion would help to make *Potentilla* monophyletic.

Gene sequences from both *Potentilla norvegica* and *P. intermedia* are present in the same major clades. This indicates that the allopolyploidy events occurred in their common ancestral lineage.

This study shows no support for species differentiation of *P. norvegica*, as previously suggested, since there was no condordance between geography and intraspecies phylogeny. In addition, the majority of the preserved specimens studied were of intermediate morphological form between the two subspecies. A more extensive study including more specimens is needed in order to determine the support for recognition of the subspecies.

Hybridization between the Argentea and Ivesioid clades may have occurred several times and given rise to the species of Wolf’s grex Rivales [25]. To better estimate when and where these hybridizations occurred, other Argentea and Rivales species of various ploidy levels should be included in future studies, such as *P. rivalis* and *P. biennis*.

## Methods

### Taxon selection

To cover the circumpolar distribution of *Potentilla norvegica* L., [60] herbarium material of one morphologically typical individual of each subspecies, ssp*. norvegica* and ssp*. hirsuta* (Michx.) Hyl., were included from Scandinavia and central Europe, in addition to two North American and one eastern Russian specimen of ssp*. hirsuta*. From the Argentea clade, species were selected if they had reported diploid populations [13], and from the Ivesioid clade the type species of *Horkelia* and *Ivesia* were selected. Low-ploidy outgroup species were selected from the Reptans, Fragarioides and Alba clades. *Potentilla intermedia* L. was also included since it shares several features with *P. norvegica*: similar morphology, weedy growth habit and assigned to grex Rivales by Wolf [25], and could therefore be suspected to have a similar evolutionary history as *P. norvegica*. All specimens included are listed in Table 2.

**Table 2:** Voucher list; low-copy markers

Taxon		Voucher	Collection site	Ploidy level	Clade	GAPCP1	GBSSI-1	DHAR2	Suffix
*Horkelia bolanderi* A.Gray		5353 (no voucher)	Cult. in Bergius Botanic Garden, Sweden	4^a^	Ivesioids	MN346800, MN346801, MN346802	–	–	
*Horkelia californica* Cham. & Schltdl.		Balls 9326 (S)	California, USA	4^a^	Ivesioids	MN346803, MN346804	MN346888, MN346889, MN346890	MN346707, MN346708	
*Horkeliella purpurascens* (S.Watson) Rydb.		Eriksson 818 (S)	Cult. in Gothenburg Botanical Garden, Sweden (from wild seeds, California, USA)	4^a^	Ivesioids	MN346805, MN346806, MN346807	–	–	
*Ivesia gordonii* (Hook.) Torr. & A.Gray		Porter 6666 (UPS)	Wyoming, USA	4^a^	Ivesioids	MN346808, MN346809, MN346810	MN346891, MN346892, MN346893, MN346894	MN346709, MN346710	
*Ivesia kingii* S.Watson		Reveal et al. 4782 (MARY)	Nevada, USA	4^a^	Ivesioids	MN346811, MN346812	–	–	
*Ivesia multifoliolata* (Torr.) D.D.Keck		Eriksson 820 (S)	Cult. in Bergius Botanic Garden, Sweden	4^a^	Ivesioids	MN346813, MN346814	–	–	
*Potentilla ancistrifolia* Franch. & Sav.	var. *dickinsii* Koidz.	2002–0674 (no voucher)	Cult. in Royal Botanic Garden Edinburgh, United Kingdom	4^a^	Fragarioides	MN346826	MN346895	MN346711	
*Potentilla argentea* L.		Flatberg, Bendiksby, Østreng Nygård (TRH)	Sør-Trøndelag, Norway	2,4,6,8	Argentea	MN346815	MN346896	MN346712	
*Potentilla aurea* L.		Persson & Eriksson NP36 (BG)	Salzburg, Austria	2	Argentea	MN346818, MN346819	–	–	115
*Potentilla aurea* L.		Persson & Eriksson NP38 (BG)	Carinthia, Austria	2	Argentea	MN346816, MN346817	MN346897, MN346898	MN346713, MN346714, MN346715	99
*Potentilla biflora* Willd. ex Schltdl.		Gabrielsen & Jørgensen (O)	Alaska, USA	2	Alba	MN346820	MN346899	MN346716	
*Potentilal brauneana* Hoppe		Persson & Eriksson NP31 (BG)	Styria, Austria	2	Argentea	MN346821	MN346900	MN346717	
*Potentilla chinensis* Ser.		Guochen-yong 20,061–446-5 (S)	Shilaizhen, Xintai Co., Shangdong Province, China	2	Argentea	MN346822, MN346823	MN346901, MN346902, MN346903	MN346718, MN346719	
*Potentilla clusiana* Jacq.		Persson & Eriksson NP29 (BG)	Styria, Austria	2,6	Alba	MN346824, MN346825	MN346904, MN346905, MN346906	MN346720, MN346721	
*Potentilla dickinsii* Franch. & Sav.		Crompton, D’Arcy & Coke 139 (E)	Cult. in Royal Botanic Garden Edinburgh, UK	2	Fragarioides	MN346827	–	–	
*Potentilla fragarioides* L.		32,074	Cult. in Botanical Garden of Bonn University, Germany	2	Fragarioides	MN346828, MN346829	MN346907, MN346908	MN346722, MN346723, MN346724, MN346725	
*Potentilla heptaphylla* L.		Persson & Eriksson NP28 (BG)	Lower Austria, Austria	2,4,6	Argentea	MN346830, MN346831, MN346832, MN346833	MN346909, MN346910, MN346911, MN346912	MN346726, MN346727	
*Potentilla hirta* L.		1962–1846 (no voucher)	Cult. in Royal Botanic Garden Edinburgh, UK	2	Argentea	MN346834	–	–	
*Potentilla intermedia* L.		Smedmark 226 (BG)	Hedmark, Norway	4,6,8	Argentea, Ivesioids	MN346835, MN346836, MN346837, MN346838, MN346839, MN346840, MN346841, MN346842, MN346843, MN346844	MN346913, MN346914, MN346915, MN346916, MN346917, MN346918, MN346919, MN346920	MN346728, MN346729, MN346730, MN346731, MN346732, MN346733, MN346734, MN346735	
*Potentilla norvegica* L.	ssp*. hirsuta* (Michx.) Hyl.	Eriksen & Töpel (GB)	Kamchatka, Russia	8,10	Argentea, Ivesioids	MN346876, MN346877, MN346878, MN346879	MN346949, MN346950, MN346951, MN346952, MN346953, MN346954, MN346955	MN346782, MN346786, MN346787, MN346788, MN346791, MN346792	112
*Potentilla norvegica* L.	ssp. *hirsuta* (Michx.) Hyl.	Gillespie, Saarela, Consaul & Bull 7439 (O)	Northwest Territories, Canada	8,10	Argentea, Ivesioids	MN346880, MN346881, MN346882, MN346883	MN346956, MN346957, MN346958	MN346793, MN346794, MN346795, MN346796, MN346798, MN346799	113
*Potentilla norvegica* L.	ssp. *hirsuta* (Michx.) Hyl.	Persson NP45 (BG)	Cult. in Bergen Museum Garden, Norway (from wild seeds, Salzburg, Austria)	8,10	Argentea, Ivesioids	MN346871, MN346872, MN346873, MN346874, MN346875	MN346945, MN346946, MN346947, MN346948	MN346773, MN346774, MN346776, MN346778, MN346779, MN346781	110
*Potentilla norvegica* L.	ssp. *hirsuta* (Michx.) Hyl.	Rouleau 6057 (UPS)	Newfoundland, Canada	8,10	Argentea, Ivesioids	MN346845, MN346846, MN346847	MN346921, MN346922, MN346923, MN346924, MN346925	MN346736, MN346737, MN346738, MN346740, MN346741	92
*Potentilla norvegica* L.	ssp. *hirsuta* (Michx.) Hyl.	Svenson AS07135 (S)	Uppland, Sweden	8,10	Argentea, Ivesioids	MN346855, MN346856, MN346857, MN346858, MN346859, MN346860, MN346861, MN346862, MN346863, MN346864	MN346931, MN346932, MN346933, MN346934, MN346935, MN346936	MN346747, MN346749, MN346750, MN346751, MN346753, MN346754, MN346755, MN346756, MN346757	96
*Potentilla norvegica* L.	ssp*. norvegica*	Eriksson 1058 (BG)	Oppland, Norway	8,10	Argentea, Ivesioids	MN346865, MN346866, MN346867, MN346868, MN346869, MN346870	MN346937, MN346938, MN346939, MN346940, MN346941, MN346942, MN346943, MN346944	MN346758, MN346759, MN346760, MN346761, MN346764, MN346765, MN346766, MN346767, MN346768, MN346769, MN346770, MN346771	97
*Potentilla norvegica* L.	ssp. *norvegica*	Gugnacka (UPS)	Kujawsko-Pomorske, Poland	8,10	Argentea, Ivesioids	MN346848, MN346849, MN346850, MN346851, MN346852, MN346853, MN346854	MN346926, MN346927, MN346928, MN346929, MN346930	MN346743, MN346744, MN346745, MN346746	95
*Potentilla reptans* L.		Salvesen 16.45 (BG)	Vest-Agder, Norway	4	Reptans	MN346884, MN346885, MN346886, MN346887	MN346959, MN346960, MN346961, MN346962	–	

Ploidy level (CCDB [13], IPCN [61]), clade [23] and Genbank accessions

^a^Based on *Ivesia baileyi var. beneolens* (A.Nelson & J.F.Macbr.) Ertter and *I. rhypara* var. *shellyi* Ertter

### Primer design

New primer pairs were designed for three low-copy nuclear markers (Table 3); GAPCP1 (glyceraldehyde-3-phosphate dehydrogenase) with primer sites in exons 11 and 14, GBSSI-1 (granule-bound starch synthase I) in exons 1 and 4 and DHAR2 (dehydroascorbate reductase 2) in exons 4 and 5. In order to find suitable primer placements, the 150 base pair long Illumina raw reads of a *Potentilla argentea* genome (putatively diploid [63]), were assembled using SOAPdenovo2 [64] on the Abel cluster (hosted by the University of Oslo, Norway). Alignments of the resulting contigs to available Rosaceae sequences at GenBank were used to screen for conserved regions in the markers. Candidate sequences were blasted in Geneious version 10.2 [65] to the *Fragaria vesca* genome published at Genbank [66] and to the *P. argentea* contigs to ensure that they would not amplify multiple regions. Annotation was based on the *F. vesca* genome (GAPCP1: XM_004306515; GBSSI-1: XM_004306569; DHAR2: XM_004307358).

**Table 3:** Primer sequences used for PCR and sequencing

Marker	Primer name	Sequence 5′-3′	Reference
GAPCP1	11F	TGT CGA CTT GAG AAG GGT GGT TC	This paper
	14R	CTT ATG CTG CCA CCA ATG CCA TG	This paper
	CGPPB5575 FWD	CAT GTG CTC TAT GAG GTC CA	[62]
	CGPPB5575 REV	ATC AGG TAT GCT GCT GAT GG	[62]
GBSSI-1	1F	TGG AG CAA GAC TGG TGG ACT TG	This paper
	4R	GCA CAA CAA GCT GAA TCT AAG TTG G	This paper
DHAR2	4F	AAG TAC ACT GAG GTA TGC TGT TC	This paper
	5R	GTT GAC TTT CGG CTC CCA TC	This paper
Cloning vector	M13–20	GTA AAA CGA CGG CCA G	StrataClone manual
	M13 Reverse	CAG GAA ACA GCT ATG AC	StrataClone manual

The *P. argentea* sequences used in this study were taken from these contigs, and were therefore not produced as the rest of the sequences (see below).

### Molecular methods

#### DNA extraction and PCR

Twenty milligrams of silica gel-dried or herbarium leaf material were extracted using the Qiagen DNeasy Plant Mini Kit (Qiagen, Valencia, CA, USA) following the manufacturer’s instructions, with the exception that the samples were left overnight at 56 °C and then allowed to lyse at 65 °C for 10 min. PCR mixtures included 2.5 μl 10x buffer (Mg^2+^ plus, 20 mM), 2 μl dNTP (2.5 mM each), 1 μl forward and reverse primers (10 μM), 0.15 μl TaKaRa Ex Taq HotStart DNA polymerase (5 U/μl) (Takara Bio, Shiga, Japan), 1–2 μl template, and ddH_2_O to add up to 25 μl. The reactions were run on a PCR C1000TM Touch Thermal Cycler (Bio-Rad Laboratories, Hercules, CA, USA). For GAPCP1, the reactions were amplified through 3 min initial denaturation at 95 °C, followed by 35 cycles of denaturation for 30 s at 95 °C, annealing for 30 s at 51 °C and extension for 1 min at 72 °C. A final extension was performed for 5 min at 72 °C. For GBSSI-1 and DHAR2, the reactions were amplified through a touch-down program with 3 min initial denaturation at 94 °C, followed by 10 cycles of denaturation for 45 s at 94 °C, annealing for 30 s starting at 55 °C and then 0.5 °C lower for each cycle, and 60s extension at 72 °C. Thirty-five cycles with a constant annealing temperature at 49 °C followed, and a final extension for 7 min at 72 °C. The reactions were checked on a 1% agarose GelRed-stained (Biotium Inc., Freemont, CA, USA) gel under UV light.

#### Cloning

Cloning of PCR products was performed on polyploids and specimens failing direct sequencing, using the StrataClone PCR Cloning kit (Agilent Technologies, Santa Clara, CA, USA) following the manufacturer’s instructions, with the exceptions that 40 and 80 μl of the transformation mixture were plated and that the reaction mixture was halved for species of lower ploidy level (4*x*). PCR reactions were performed on positive transformants with primers M13–20 and M13 reverse (as found in the manual) together with Ex-Taq HS polymerase as described above. Amplification started with an initial denaturation for 10 min at 94 °C, followed by 35 cycles of denaturation for 45 s at 94 °C, annealing for 45 s at 55 °C and extension for 3 min at 72 °C. A final extension was performed for 10 min at 72 °C. PCR products were then checked on a 1% agarose gel.

#### Purification and sequencing

All PCR products were purified using the Exo-Sap method [67]. The number of clones sequenced corresponded to 95% probability of finding all gene copies, that is at least 6 clones for tetraploids, 11 clones for hexaploids, 16 clones for octoploids and 21 clones for decaploids (Lundberg et al., unpublished). Two species of *Ivesia* have been reported to be tetraploids [61], and therefore the Ivesioid species included in this study were also treated as such. The samples were prepared using a BigDye Terminator Cycle sequencing kit (Applied Biosystems, Waltham, MA, USA) and run on an ABI 3730XL DNA Analyser (Applied Biosystems). For DHAR2, some samples were sent to Macrogen Sequencing Service (Amsterdam, The Netherlands) after purification. All other molecular labwork was carried out at the Biodiversity Laboratories (DNA Section) at the University of Bergen.

### Sequence treatment and alignment

For each marker, forward and reverse reads for each specimen or clone were assembled using PreGap4 and Gap4 of the Staden Package [68]. Automatic alignment of each cloned species separately (and specimen, in the case of *P. norvegica*) was performed in AliView v. 1.18 [69] using MUSCLE [70]. Putative PCR errors were corrected and identical sequences were removed. An alignment with all *P. norvegica* specimens was then performed, in order to remove identical sequences shared between individuals.

To detect PCR recombinants, the alignments of cloned specimens were loaded into SplitsTree v. 4.14.4 [71]. Sequences identified as putative PCR recombinants had no, or very short, individual edges and long, parallel, connecting edges to their parental sequences [72]. All remaining sequences were automatically aligned together in AliView followed by manual adjustments.

### Phylogenetic analyses

#### Model testing

Substitution model testing was performed on each marker with PartitionFinder2 [73], with GAPCP1 and GBSSI-1 divided into subsets of introns and the three codon positions, under the BIC and AICc criteria for the models available in MrBayes. DHAR2 was not divided into subsets, since the amplified region almost exclusively consists of the intron between exons 4 and 5.

#### Indel coding

Indels found in two or more sequences were manually coded according to the Simple Indel Coding method as present (1), absent (0) or inapplicable (N) [74].

#### Bayesian inference

Bayesian Inference analyses were run for each marker separately in MrBayes v. 3.2.6 [75, 76], using the Metropolis Coupled Markov Chain Monte Carlo algorithm [77], including one cold chain and three heated chains for each of two runs. Division of the alignments into subsets and assignment of models were coded according to the results from PartitionFinder2 (Table 1). The Mk model [78] was applied for the indels, where the likelihood prior Coding and rate prior were set to variable. The analyses were run for 5 million generations for GAPCP1 and GBSSI-1, and 7.5 million generations for DHAR2, with sampling from the chain every 1000th generation and with a burnin of 20%. An analysis was accepted if the standard deviation of split frequencies was below 0.01, the chain swap was between 20 and 80% [79] (McGuire et al. 2007), no trend was seen in the overlay plot and the Potential Scale Reduction Factor [80] values had reached 1.0 for all parameters. A clade was fully accepted if its Bayesian posterior probability was 0.95 or higher. In order for the DHAR2 analysis to converge, 13 *P. norvegica* sequences and one *P. intermedia* sequence that were suspected to cause problems had to be removed. These were identified by inspecting the whole dataset in SplitsTree. PartitionFinder2 and MrBayes were run at the CIPRES Science Gateway [81].

#### Maximum likelihood

Maximum Likelihood analyses were performed in RAxML version 7.2.8 [82, 83]. under the GTR + G (nucleotides, DNA) [84] and Mk (indels, MULTI) [71] models with 1000 rapid bootstrap replicates [85]. A clade was fully accepted if its Bootstrap support was 75 or higher.

#### Rooting and tree graphics

The resulting consensus trees from the BI and ML analyses were inspected using FigTree version 1.4.1 [86] and rooted on *P. biflora* and *P. clusiana* Jacq. of the Alba clade. The Alba clade is the sister clade to the rest of the species included in this study [18, 23]. All branches with posterior probabilities below 0.8 were collapsed in Mesquite version 3.10 [87]. The layouts were further edited using GIMP version 2.8.10 (www.gimp.org) and Inkscape version 0.48 (www.inkscape.org).

#### Control analyses

To ensure that no gene copies were incorrectly discarded as PCR recombinants, all unique sequences of the Ivesioids (*Horkelia*, *Horkeliella* and *Ivesia*), *P. intermedia* and *P. norvegica* were subjected to an ML analysis each (without coded indels), together with a reduced dataset of the species representing the larger clades seen in the gene trees.

#### Multispecies coalescent analysis

Due to initial results from the BI and ML analyses showing somewhat different topologies for the different markers, some species were subjected to a Multispecies Coalescent analysis [88] in BEAST v. 1.8.0 [89] at CIPRES [81], in order to create a species tree as a backbone reference. Two datasets were created, one with the three low-copy markers only, and one with the low-copy markers in combination with three chloroplast regions from previous studies (trnL-F, trnC-ycf6 and trnS-ycf9) (Table 4) [18, 44, 90]. Substitution model testing was performed in PartitonFinder2 on each region, not accounting for codon positions. Two clock models were tested; strict and relaxed uncorrelated log normal [91]. For each of these, two tree priors were tested; a birth-death process [92] and a birth process [93]. The analysis of the dataset with low-copy markers only was run for 50 million generations with sampling every 1000th generation, and the combined dataset for 150 million generations with sampling every 1000th generation. To test the fit of the models to the data, path sampling and stepping-stone sampling [94, 95] were performed with 50 steps, each with a length of 1 million iterations for the low-copy marker dataset, and 150 steps with a length of 1 million iterations for the combined dataset. Log marginal likelihood differences larger than three were considered significant [96]. The analysis with the models best fit to the data was run two independent times, and the results were inspected using Tracer v. 1.7.1 [97]. In order to test if the prior, rather than the data, was driving the results, an additional run with sampling from prior only was performed. The tree files were then combined using TreeAnnotator of the BEAST package with a burnin of 20% of each run.

**Table 4:** Voucher list; chloroplast markers

Taxon	Voucher	Collection site	Ploidy level	Clade	trnL-trnF	trnS-ycf9	trnC-ycf6
*Horkelia californica* Cham. & Schltdl.			4^a^	Ivesioids	FR872958	–	–
*Horkeliella purpurascens* (S.Watson) Rydb.	Ertter 4980 (UC)	California, USA	4^a^	Ivesioids	GQ384737	GQ384569	GQ384891
*Ivesia gordonii* (Hook.) Torr. & A.Gray	Huber 1182 (MO)	Utah, USA	4^a^	Ivesioids	GQ384725	GQ384557	–
*Potentilla argentea* L.	Gregor (HEID)	Rhineland-Palatinate, Germany	2,4,6,8	Argentea	GQ384652	GQ384485	GQ384820
*Potentilla argentea* L.	Dobeš (HEID)	Lower Austria, Austria	2,4,6,8	Argentea	GQ384665	GQ384497	GQ384833
*Potentilla argentea* L.	Krämer et al., Botanical Garden Bonn	North Rhine-Westphalia, Germany	2,4,6,8	Argentea	GQ384675	GQ384507	GQ384843
*Potentilla aurea* L.	Dobeš (HEID)	Tyrol, Austria	2	Argentea	GQ384667	GQ384499	GQ384835
*Potentilla aurea* L.	Paule (HEID)	Julian Alps, Slovenia	2	Argentea	GQ384673	GQ384505	GQ384841
*Potentilla biflora* Willd. ex Schltdl.	S. Kharkevich, T. Buch	Magadan Oblast, Russia	2	Alba	GQ384682	GQ384514	GQ384850
*Potentilal brauneana* Hoppe	Dobeš (HEID)	Tyrol, Austria	2	Argentea	GQ384668	GQ384500	GQ384836
*Potentilla chinensis* Ser.	Zhechai (CM)	China	2	Argentea	KT991783	–	–
*Potentilla clusiana* Jacq.	Leopoldinger, Univerity of Salzburg Botanical Garden	Upper Austria, Austria	2,6	Alba	GQ384640	GQ384473	GQ384808
*Potentilla heptaphylla* L.	Dobeš (HEID)	Lower Austria, Austria	2,4,6	Argentea	GQ384666	GQ384498	GQ384834
*Potentilla hirta* L.	Dobeš (HEID)	Alpes-Maritimes, France	2	Argentea	GQ384634	GQ384467	GQ384802
*Potentilla reptans* L.	Botanical Garden Nantes Mairie	Pays de la Loire, France	4	Reptans	GQ384638	GQ384471	GQ384806

Ploidy level (CCDB [13], IPCN [61]), clade [23] and Genbank accessions

^a^Based on *Ivesia baileyi var. beneolens* (A.Nelson & J.F.Macbr.) Ertter and *I. rhypara* var. *shellyi* Ertter

### Morphological study

**Fig. 6:**
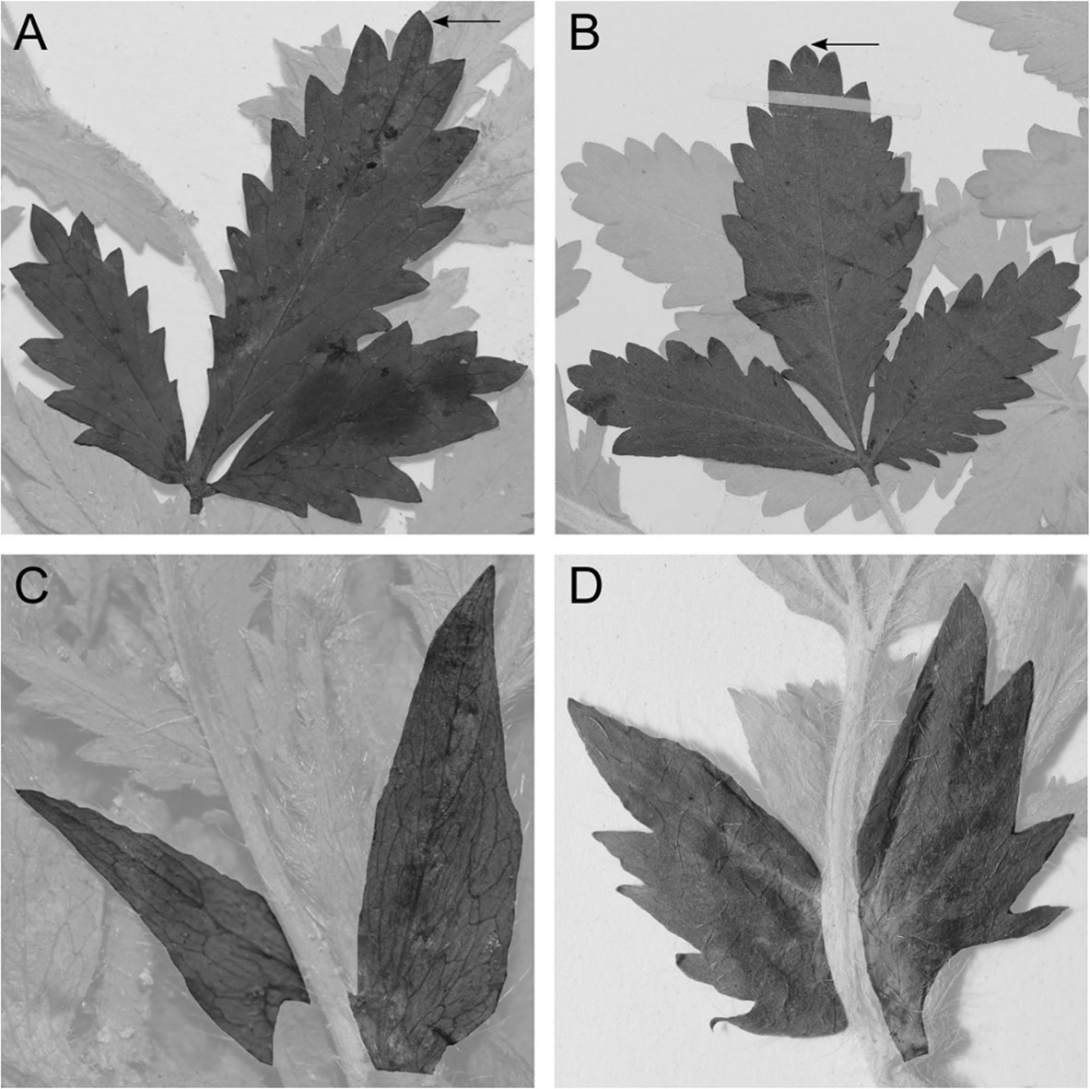
Images of *Potentilla norvegica* leaves and stipules, illustrating the typical characters for ssp. *norvegica* and ssp. *hirsuta*: Leaflet and tooth shape (**a** and **b**) and stipule dentation (**c** and **d**)*.* The specimen in **a** and **c** was collected by Gugnacka s.n. (UPS) in Poland, here denoted individual 95. The specimen in **b** and **d** was collected by Rouleau nr. 6057 (UPS) in Canada, here denoted individual 92

*Potentilla norvegica* specimens were inspected at, or on loan from, the herbaria of Stockholm (S), Uppsala (UPS) and Gothenburg (GB) in Sweden, and the herbaria of Bergen (BG) and Oslo (O) in Norway. They were used to study the defining characters of the two *P. norvegica* subspecies (ssp*. norvegica* and ssp*. hirsuta*); leaflet form, leaflet dentation and stipule dentation [29, 38, 98] (Fig. 6 and Table 5).

**Table 5:** Morphological characters used to differentiate between the two subspecies of *Potentilla norvegica*

	ssp. *norvegica*	ssp. *hirsuta*
**Basal leaflets**	oblanceolate	obovate
	acute terminal tooth	obtuse terminal tooth
	long terminal tooth	short terminal tooth
**Stipule teeth**	0–3	2 – several

## Data Availability

Most specimens included are deposited at herbaria (see Table 1). Vouchers are missing for a few of the specimens obtained from botanical gardens. The DNA sequences are deposited at GenBank under the accession numbers [MN346707-MN346962].
